# Determinants of access to HIV testing and counselling services among female sex workers in sub-Saharan Africa: a systematic review

**DOI:** 10.1186/s12889-018-6362-0

**Published:** 2019-01-05

**Authors:** Soori Nnko, Evodius Kuringe, Daniel Nyato, Mary Drake, Caterina Casalini, Amani Shao, Albert Komba, Stefan Baral, Mwita Wambura, John Changalucha

**Affiliations:** 1 Department of Sexual and Reproductive Health, National Institute for Medical Research, Isamilo Road, P.O Box 1462, Mwanza, Tanzania; 2 Sauti Program, Jhpiego Tanzania - an affiliate of Johns Hopkins University, P.O Box 9170, Dar es Salaam, Tanzania; 3 Key Populations Program, Center for Public Health and Human Rights, Department of Epidemiology, Johns Hopkins Bloomberg School of Public Health, E7146, 615 N. Wolfe Street, Baltimore, MD 21205 USA

**Keywords:** Determinants, Access, HIV testing and counselling, Female sex workers, Sub-Saharan Africa

## Abstract

**Background:**

HIV testing and counselling (HTC) is an essential component for HIV prevention and a critical entry point into the HIV continuum of care and treatment. Despite the importance of HTC for HIV control, access to HTC services among female sex workers (FSWs) in sub-Saharan Africa (SSA) remains suboptimal and little is known about factors influencing FSWs’ access to HTC. Guided by the client-centred conceptual framework, we conducted a systematic review to understand the facilitators and barriers influencing FSWs in SSA to access HTC services.

**Methods:**

A systematic search was conducted in MEDLINE, POPLINE and Web of Science databases for literature published between January 2000 and July 2017. References of relevant articles were also searched. We included primary studies of any design, conducted in SSA and published in the English language. Studies conducted in multi-sites inclusive of SSA were included only if data from sites in SSA were separately analysed and reported.

Similarly, studies that included other subpopulations were only eligible if a separate analysis was done for FSWs. This review excluded papers published as systematic reviews, editorial comments and mathematical modelling. The protocol for this review is registered in the *Prospective Register of Systematic Reviews* (PROSPERO), registration number CRD42017062203.

**Results:**

This review shows that factors related to approachability, acceptability, availability, affordability and appropriateness of the services are crucial in influencing access to HTC services among FSWs in SSA. These factors were mediated by individual attributes such as HIV risk perceptions, awareness of the availability of HTC, and perceptions of the importance and quality of HTC services. The decision to utilise HTC was predominantly hampered by discriminatory social norms such as HIV stigma and criminalisation of sex work.

**Conclusions:**

FSWs’ access to HTC is facilitated by multiple factors, including individual awareness of the availability of HTC services, and perceived quality of HTC especially with regard to assured confidentiality. Concerns about HIV stigma and fear about discrimination due to community intolerance of sex work acted as major barriers for FSWs to seek HTC services from the facilities offering health services to the general population.

**Electronic supplementary material:**

The online version of this article (10.1186/s12889-018-6362-0) contains supplementary material, which is available to authorized users.

## Background

Globally, female sex workers (FSWs) are at heightened risk of acquiring HIV compared to other females of reproductive age [1–3]. According to a systematic review and meta-analysis study, the risk of acquiring HIV across SSA is 13 times higher for FSWs [1]. Equally, HIV prevalence among FSWs in the region is comparatively higher [1, 4]. In some settings of SSA countries, HIV prevalence among FSWs has been reported to be as high as 40% [1].

HIV transmission in SSA is attributable mainly to heterosexual relationships [5–7], and sex work plays a vital role in HIV transmission [7–10]. Sexual relationships between FSWs and other members from the general  population potentiate HIV transmission since FSWs exhibit high risk sexual behaviours such as unprotected sexual intercourse and high rates of partner change [11]. A modelling study on population attributable fraction (PAF) estimates of HIV infections estimated that, the PAF of HIV infections among women of reproductive age in the general population due to female sex work in SSA was 18% in 2011 [12]. Nevertheless, due to structural barriers in most SSA countries, FSWs are less likely to access HIV prevention and treatment services [13]. Thus, to achieve the UNAIDS goal of ending the HIV epidemic as a public health threat by 2030,the emphasis has been to ensure equitable access of FSWs and other key populations (KPs) to HIV prevention services including HTC [14].

HTC is an essential component for HIV control. Apart from allowing individuals to know their HIV status and providing an entry point to the HIV care and treatment for the people infected with HIV [15, 16], HTC has been shown to influence positive behaviour change for those found to be infected with HIV [17–19]. Recognising the importance of HTC in HIV control, the World Health Organization (WHO) has developed Consolidated Guidelines for HIV prevention, diagnosis, treatment and care among key populations including FSWs, recommending for routine HTC [18].

To optimise delivery of HTC and achieve equitable access to HTC services, HIV prevention programs across SSA have used various strategies, including facility-based approaches, stand-alone clinics, and community approaches, e.g. mobile clinics, outreaches, and moonlighting [20]*.* Despite the existence of multiple strategies to deliver HTC, FSWs’ access to these services remain unacceptably low [21, 22].

**Fig. 1:**
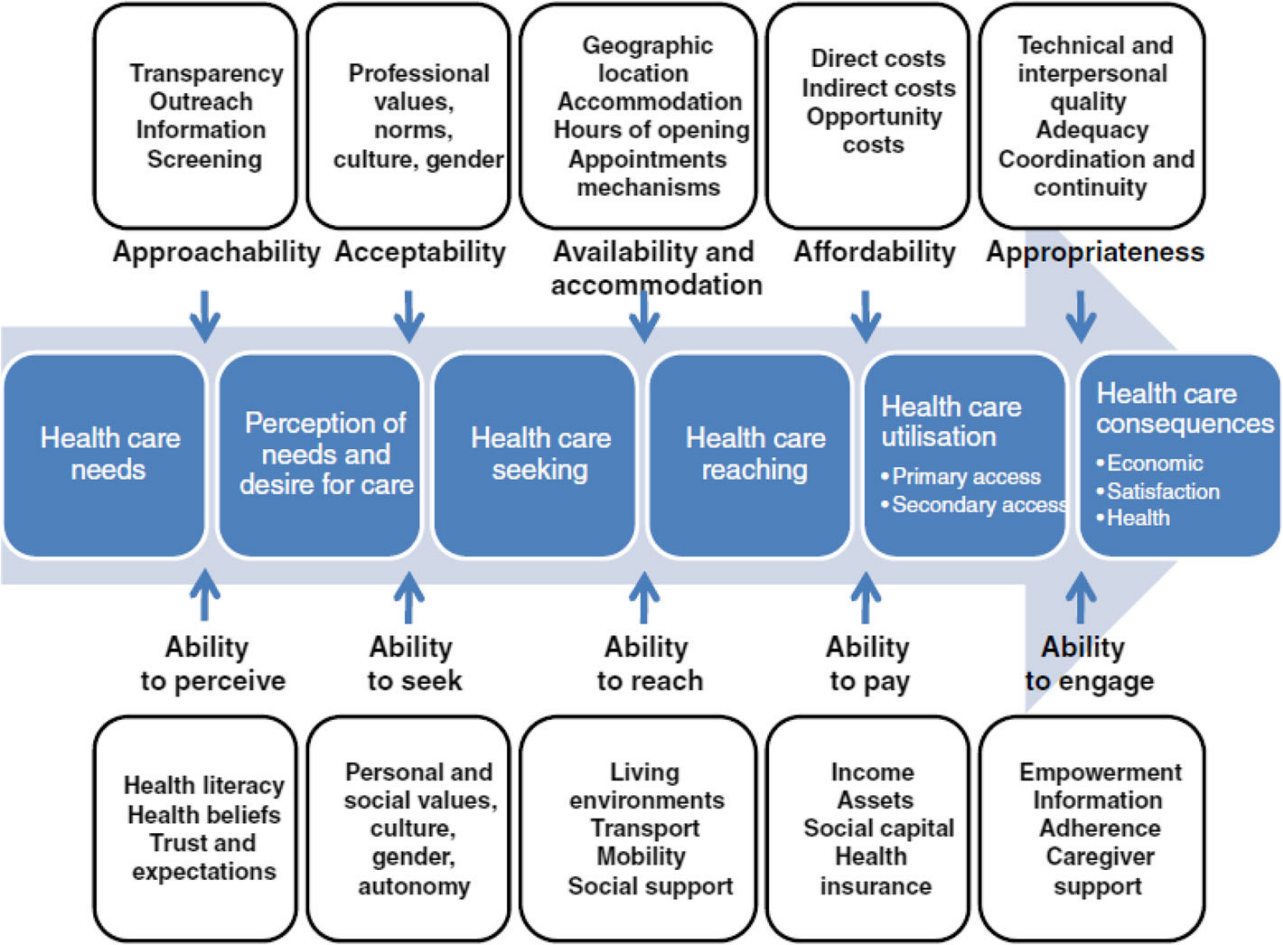
A conceptual framework of access to health care developed by Levesque et al. Source: Levesque et al. International Journal for Equity in Health 2013, 12:1

WHO defines access to health care as: “the ability of an individual or a defined population to obtain or receive appropriate health care” [23]. There are other variant versions of the definition for “access to health care services” [23–27], however in common, access is defined as a product by *demand* and *supply* sides of health care services. According to Levesque and colleagues, access to services is the “possibility for a person or member of family to **identify** healthcare needs, to **seek** health care services, to **reach** health care resources, to **obtain** or use health care services, and to actually be **offered** services appropriate to the needs for care” [26]. This description underscores the role of the interplay between demand (individual, community and population levels attributes) and supply (e.g. health systems, programs, institutions, organisations and providers) on access to services. Furthermore, using a demand-supply dichotomy, Levesque and colleagues prescribe five dimensions of “access to care” namely; “*approachability*”, “*acceptability*”, “*availability and accommodation*”, “*affordability*” and “*appropriateness*” [26]. Levesque and colleagues define *approachability* as that state where individuals with health needs are aware of the existence of services, and the services are reachable and have an influence on their health [26]. *Acceptability* relates to the socio-cultural factors that regulate the probability of service acceptance and care seeking by the individuals, and it also includes issues on equity during access [26]. *Availability and accommodation* relate to services being obtained easily and timely, it relates to services being adequate in terms of amount, distribution and organisation [26]. *Affordability* refers to the economic ability of individuals to cater for their health services [26]. This includes financial and time spent on health services; whereas *appropriateness* refers to how the services provide answers to the clients’ needs, this includes the types and quality of the services provided [26]. This framework assumes that access to services is a result of interaction between five dimensions, namely: i) ability to perceive, ii) ability to seek, iii) ability to reach, iv) ability to pay and v) ability to engage (Fig. 1) [26].

This review sets to understand the facilitators and barriers to accessing HTC among FSWs in sub-Saharan Africa using the client-centred conceptual framework developed by Levesque and colleagues [26]. Understanding facilitators and barriers to accessing HTC help to fill the knowledge gap and guide HIV programmers to address challenges in HTC service delivery.

## Methods

This systematic review was conducted to understand access to HTC among FSWs using Levesque theoretical framework. The protocol for this review is registered in the Prospective Register of Systematic Reviews (PROSPERO), registration number CRD42017062203. Preferred Reporting Items for Systematic Reviews and Meta-Analyses (PRISMA) guidelines were followed (Additional file 1).

### Search strategy

A systematic search was conducted in MEDLINE, POPLINE and Web of Science databases for literature published between January 2000 and July 2017. References of relevant articles were also searched. MEDLINE search was done using text words and medical sub-headings (MeSH terms) and comparable terms were used for POPLINE and Web of Science databases. The search was divided into four parts: **topic**, **population**, **intervention** and **context (regional coverage)**. For these four parts, the search terms were combined to limit the number of publications obtained. The search terms used for ***topic*** were; ‘Human Immunodeficiency virus’, ‘HIV’, ‘HIV-1’, ‘HIV-2’, ‘HIV type 1’, ‘HIV type 2’ and ‘acquired immunodeficiency syndrome’. The ***population*** terms included; ‘sex work*’, ‘female sex work*’, ‘commercial sex work*’, ‘prostitut*’, ‘women who exchange sex for money’ and ‘women who sell sex’. The ***intervention*** included terms such as; ‘voluntary HIV testing’, ‘HIV testing and counselling’, ‘HIV diagnosis’ and ‘HIV screening’. The included literatures were all from sub-Saharan Africa ***context***. The regional or geographical coverage (***context)*** terms included the terms: ‘Africa south of the Sahara’, ‘Africa’, South’, ‘Sahara’, ‘Africa south of the Sahara’, ‘sub’, ‘Saharan’, ‘sub-Saharan Africa’. Details on the search strategy are provided in Additional file 2.

### Eligibility criteria

**Fig. 2:**
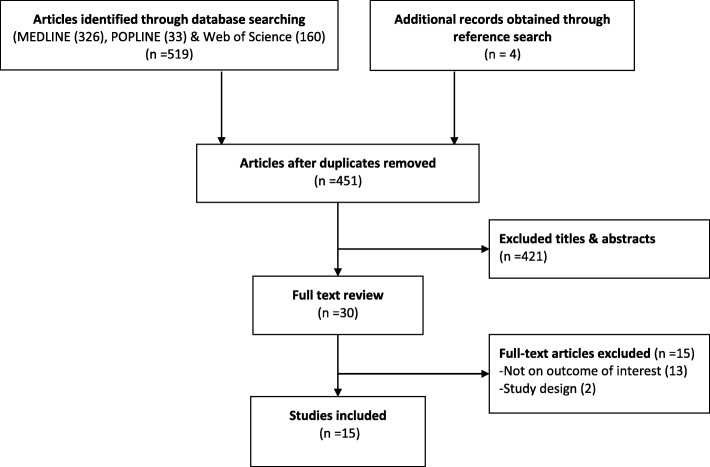
PRISMA Flow diagram of article selection

The UNAIDS defines sex worker as female, male or transgender who receives money or goods in exchange for sexual services [28]. However in this study, we limited our search to papers addressing females who exchange sex for money or other commodities either regularly or occasionally. We limited our search to papers published between January 1st, 2000 and July 31st, 2017. We restricted the search to begin in the year 2000 mainly because most of the countries in SSA started to incorporate HIV testing in their national HIV control programs from early 2000 [29]. Moreover, the advancement in rapid HIV testing technologies and the establishment of the Global Fund in 2001 and the United States President’s Emergency Plan for AIDS Relief (PEPFAR) in 2003 led to the expansion of both HTC and antiretroviral therapy in SSA [30]. Only studies conducted in sub-Saharan Africa and published in English language were eligible for this review. Because of the limited number of published studies on this topic, we included primary studies of any design reporting on access to HTC among FSWs. Studies conducted in multi-sites including sites from SSA were included only if data from sites in SSA were separately analysed and reported. In addition, studies that included other subpopulations were only included if a separate analysis was done for FSWs. Since this systematic review aims to analyse studies using primary data, we excluded papers published as a systematic review, editorial comment and mathematical modelling. Figure 2 summarises different phases of the study selection process.

### Quality assessment

The methodological and reporting quality of included studies were assessed using an adapted tool (Additional file 3), incorporating items from the Cochrane Collaboration qualitative study critical appraisal tool [31], the consolidated criteria for reporting qualitative studies (COREQ) tool [32] as well as the strengthening reporting of observational studies in epidemiology (STROBE) tool [33]. The assessment tool included criteria such as clearly documented eligibility criteria, sufficient description of methods and results, sampling strategies, internal validity (credibility for qualitative studies) and generalizability (transferability for qualitative studies) of the findings. This assessment was performed independently by two authors. A discrepancy in the rating of the quality between the authors was resolved involving a third author during the discussion. A score of at least 70% was rated as high, 40–69% as moderate and below 40% as low quality. However, we did not exclude any study from our analysis to gain a broader understanding of the insights regarding barriers and facilitators of HTC uptake among FSWs from all the studies regardless of the methodological or reporting weaknesses.

### Records screening and data extraction

Publications were organised and screened using the *Covidence* systematic review toolkit*.* After the search, all identified records were imported into *Covidence* for screening. The *Covidence* toolkit automatically removed some of the duplicate records while other records were removed during a later stage when screening the full articles. Later, two reviewers (SN & EK) independently screened titles and abstracts to include those that potentially included information on barriers and, or facilitators of access to HTC among FSWs. Titles and abstracts which were considered as relevant, their full texts were retrieved for review. Discrepancies in screening results of the titles and abstracts were resolved by including the discrepant article for the full-text screening.

Full-text reviews were independently done by the first two authors (SN & EK). Any disagreement on the eligibility of the full papers included was resolved through discussion with a third reviewer (DN). Finally, information from the eligible studies was extracted using a standardised data extraction form which included authors, publication year, study sites, study aim and design. See Table 1.

**Table 1:** Characteristics of selected articles

Author/ citation	Country	Study aim	Study design/type of data
Aho et al., (2011) [35]	Guinea	To describe the acceptability and outcomes of HTC among a stigmatised and vulnerable group.	Baseline: Interview / FGDs & survey (*n* = 421) plus HIV screen. Follow-up survey (*n* = 223) plus HIV test; Sampling: attendees at private or public centres providing healthcare services.
Ameyan et al., (2015) [36]	Ethiopia	To explore the barriers to utilising HTC facilities and identify the motives and motivations of FSWs who seek HTC.	Cross-sectional qualitative study; In-depth interviews with FSWs (20); Purposive sampling.
Batona et al., (2015) [42]	Benin	To identify psychosocial factors associated with the intention to be HIV tested.	Cross-sectional study; Questionnaire survey (*n* = 450); Cluster sampling;
Dugas et al., (2015) [43]	Benin	To examine the potential of 3 different categories of outreach intervention to increase the use of testing services in Benin.	Cross-sectional ethnographic study (semi-structure interviews (*n* = 66); Purposive sampling
Langa et al., (2014) [44]	Mozambique.	To assess HIV risk perception, sexual behaviour and treatment seeking among FSWs.	In-depth semi-structured interviews & focus group discussions; *n* = 236 FSWs from three sites; Snowball sampling approach.
Nakanwagi et al., (2016) [45]	Uganda	To identify the facilitators and barriers to linkage to HIV care among FSWs who tested positive to design appropriate HIV interventions for this key population group.	Cross-sectional qualitative study; In-depth interviews (*n* = 28 FSWs); Purposively selection of FSWs accessing HIV services from mobile outreaches.
Scorgie et al., (2013) [46]	Kenya; Uganda; Zimbabwe; South Africa	To examine experience of key populations in seeking public and private healthcare and barriers to accessing these services.	Cross sectional qualitative study; In-depth interviews (*n* = 55) & focus group discussions; Snowball sampling.
Lafort et al., (2016) [47]	Kenya, Mozambique; South Africa	To assess where FSW go for care in different settings, and what motivates their choice.	Multi-site cross-sectional survey (South Africa (*n* = 400), Tete, Mozambique (*n* = 308), Mombasa, Kenya (*n* = 400); Respondent-driven sampling (RDS).
Lafort (2016) [48]	Mozambique	Assess factors that facilitate or hinder utilisation of HIV and sexual and reproductive health services among FSWs.	Cross-sectional survey (*n* = 311); together with In-depth interviews & Focus Group Discussion (FGDs); Respondent-driven sampling (RDS)
Luseno et al., (2009), [38]	South Africa	To identify factors that facilitates or hinders HIV testing among South African women with high risk for HIV infection.	Randomised trial; baseline data (*n* = 425); Participant recruited through targeted street outreach.
Mulongo et al. (2015) [49]	Democratic Republic of Congo	To assess the impact of community-based prevention and HIV counselling and testing approaches in reaching FSWs with prevention messaging and treatment options.	Participatory appraisal including follow-up testing, care, and treatment to HIV positive individuals; Snowball sampling
Renzaho et al., (2009) [37]	Tanzania	To explore the knowledge and practices about HIV among female sex workers (CSWs) and assess the contextual dynamics that prevent safer sexual behaviours.	Semi-structured face-to-face interviews (=54) & discrete focus group discussions (*n* = 26); Snowball sampling approach.
Chanda et al., (2017) [41]	Zambia	To explore perceived barriers and facilitators of HIV testing among FSWs	Cross-sectional; focus groups (*N* = 5), total participants = 40)
Wanyenze et al., (2017) [39]	Uganda	To explore barriers to HIV service access and opportunities for increasing access to services	Cross-sectional study; focus group discussion (FGD) (*n* = 24), total participants = 190
Nyblade et al., (2017) [40]	Kenya	To explore the relationship between healthcare worker sex-work stigma and HIV counselling and testing & utilisation of non-HIV health services among female and male sex workers	Cross-sectional survey; snowball sample of 497 FSWs

### Data analysis and synthesis

All information extracted was synthesised using narrative synthesis approach. Narrative synthesis offers an opportunity to describe review findings in terms of themes, words and, or text [34]. Factors that affected access to HTC among FSWs were sorted, and categorised according to the five dimensions of access to health care developed by Levesque and colleagues [26].

## Results

Five hundred twenty-three records were identified from the search. Titles and abstracts were screened from 451 unique records and 421 records excluded. Thirty articles remained for full-text review. Of the remaining articles, 15 papers were excluded from the analysis, among which 13 were not focused on the outcome of interest and others due to being systematic reviews. Figure 2 illustrates the search result and the screening process. Only 15 papers met all criteria [35–49] for inclusion in this synthesis as shown in Table 1. From the quality assessment tool, two studies were rated high [40, 42], 12 studies moderate and one study was rated low quality [43].

Fifteen studies, spanning 11 countries met the inclusion criteria. Of those, eight studies were conducted among FSWs alone [35, 37, 39, 41, 42, 46–48], while four studies included other study participants, e.g. health providers and clients of FSWs [36, 43–45]. Three studies included FSWs and other key populations, such as men who have sex with men (MSM) [40, 49] and abusive alcohol and cannabis users [38].

Seven papers presented qualitative data from ethnographic studies (mostly collected through in-depth interviews, or a combination of in-depth interviews and participatory group discussions) [36, 39, 41, 43–45, 49]. Four papers were from quantitative studies collected through cross-sectional surveys without comparison groups [40, 42, 47, 48], and three other papers presented data from mixed methods [35, 37, 46]. Only one paper presented data from a randomised controlled trial [38]. Table 1 describes the characteristics of studies included in this review.

### HTC service delivery approaches

Studies included in this review have utilised a variety of service delivery approaches for HTC services provision to FSWs. These approaches include facility-based and community-based HTC service delivery approaches. Facility-based HTC was offered to FSWs through public and private health facilities [35, 47–49]. Facility-based HTC delivery to FSWs was implemented either through general health care clinics including antenatal clinics [35, 48, 49] or stand-alone clinics and drop-in-centres [47–49]. Community-based approaches reported in the articles included outreach and mobile approaches including mobile moonlight clinics [43, 45, 49] as well as home and work-based HIV testing services [43]. Other studies utilised a combination of these approaches for HTC delivery to FSWs. None of the studies reported on FSWs' experience with HIV self-testing.

### Determinants of access to HTC

Using Levesque’s theoretical framework, most publications addressed a combination of dimensions of access to health services. For example, some studies described socio-cultural aspects to HTC access and uptake among FSWs (related to acceptability dimension) and how the HTC needs of the FSWs are met (appropriateness of the services) [35, 37, 39, 40, 45, 46, 49]. While, other studies described the awareness of the existence of HTC services by FSWs (approachability dimension) and the quality of the services provided [35, 39, 44, 45, 48].

Majority of papers described acceptability of the HTC services among FSWs [35–39, 41–46, 49]. Table 2 summarises the thematic focus of studies included in the review.

**Table 2:** The thematic focus of selected studies (based on conceptual framework developed by Levesque et al.)

Study ID	Reported dimensions of access to voluntary HTC				
	*approachability*	*acceptability*	*Availability & accommodation*	*affordability*	*appropriateness*
Aho et al., (2011) [35]	√	√			
Ameyan et al. (2015) [36]	√	√	√	√	√
Batona et al., (2015) [42]	√	√			
Dugas et al.,(2015) [43]	√	√			
Langa etal., (2014) [44]		√		√	
Nakanwagi et al., (2016) [45]		√	√		√
Scorgie et al., (2013) [46]	√	√	√	√	√
Lafort et al., (2016) [47]			√	√	√
Lafort et al., (2016) [48]			√	√	√
Luseno et al., (2009), [38]	√	√			
Mulongo et al., (2015) [49]	√	√	√		√
Renzaho et al., (2009) [37]		√	√		
Chanda et al., (2017) [41]	√	√	√		
Wanyenze et al., (2017) [39]	√	√	√	√	√
Nyblade et al., (2017) [40]					√

### Approachability of HIV testing and counselling services

Awareness of the existence and importance of HTC services, attitude toward HTC services and perceptions of individual HIV risk influence access of HTC among FSWs. Conversely, low awareness of the importance, availability and locations where HTC services can be found contribute to poor uptake of services [36, 39, 41–43, 46, 49]. In a study conducted in the Democratic Republic of Congo (DRC), FSW peers were used to improve awareness of service availability and location which improved uptake of HTC [49].

Three papers discussed the role of risk perception in shaping FSWs decision to access and use HTC services [35, 38, 42]. Suspicion on their previous sexual behaviour or their partner’s sexual behaviour was a common determinant on how FSWs perceived their own risk of HIV infection. FSWs who perceived themselves as being at a higher risk of HIV infections were more likely to seek HTC than those with low-risk perception [35, 38, 42]. In addition, a study conducted in Guinea found that FSWs who perceived to be at high risk of HIV infection, were more likely to return for their HIV test results than those who felt they were at a moderate or low risk (OR 2.7; 95% CI [1.3–5.9]) [35]. According to this study, experiencing HTC among FSWs was associated with increased likelihood of returning for their test results and accepting follow up HTC [35]. One study indicated that FSWs who perceived themselves to be at low risk of HIV infection were willing to go for HTC service only when they felt sick [36].

### Acceptability of HIV testing and counselling services

Socio-cultural and economic factors also play a role in influencing access to HTC among FSWs.

Studies show that FSWs peers can positively influence the uptake of HTC through encouragement and motivation to take the test [35, 36, 39, 41–43, 45, 49]. Studies report strong motivation to access HTC services among FSWs coming from their peers. The literature also shows that the use of trained FSW peers in creating demand for HTC can increase the uptake of these services [36, 49]. In a study conducted in the DRC, the use of FSW peers led to a significant improvement in the uptake of HTC services [49]. However, one study cautioned on poor social cohesion among FSWs due to high mobility and violence [43].

The majority of the reviewed studies reported stigma associated with HIV and sex work to be the leading barrier for FSWs to access HTC. Seven out of 15 papers that were reviewed reported that HIV stigma reinforced fear to undertake HTC [38, 39, 41–44, 46]. Being a sex worker was automatically associated with having HIV and therefore reinforced self-stigmatisation among FSWs [41]. For example, one study reported that some FSWs perceived themselves to be already HIV positive and therefore, did not consider HIV testing to be meaningful [41]. FSWs feared that undertaking HIV test may have a negative consequence especially if their HIV test results are known to other people. Seven studies reported that FSWs were concerned about potential consequences such as stigmatisation, discrimination, social exclusion and work termination [38, 39, 41–44, 46]. One study reported that FSWs feared that if their HIV positive status is known to other people, it may cause them to lose sexual clients and income [36]. Other consequences reported included banishment from the worksite and verbal abuse [35].

Although in most studies the main reasons cited for HTC acceptance was the wish to know individual HIV status, there were three papers which reported that some of the managers and owners of recreational facilities forced FSWs to be tested, curtailing their free decision making [35, 36, 46]. One of the papers even reported a case where certain managers and owners of recreational facilities forced FSWs to disclose their HIV test results to them if they were to continue operating from their facilities [35].

### Availability and accommodation of HIV testing and counselling services

Service supply-side factors are essential in influencing access and uptake of HTC among FSWs. HTC services availability, location, adequacy of facilities and test kits and service organisation have a potential to improve access among FSWs [36, 37, 41, 45, 47].

HTC service location was found to be an important reason that influences service uptake among FSWs [36, 45, 47]. However, there were mixed findings of the role of proximity to health facilities offering HTC services, whereas, while some FSWs preferred HTC services closer to their residencies, others preferred services far from their localities. Some studies reported that short distance to the HTC facility increases the use of HTC services among FSWs [36, 45, 47]. FSWs who preferred to seek HTC services from nearby health facilities reported that the short distance saves time and transport costs [45]. Conversely, other papers suggested that some FSWs fear to use nearby facilities for HTC services because of perceived and experienced lack of privacy, breach of confidentiality, and concern about stigmatisation at health facilities [36, 37, 39, 45, 46]. For these reasons, some FSWs opted to seek HTC services from health facilities located in areas far away from their place of residence or worksite [36, 37, 39, 45, 46].

The organisation of HTC service including working hours also contributes to determining FSWs' access to HTC [36, 39, 46, 47, 49]. Poor HTC service organisation, characterised by long waiting time and lack of privacy, discourages FSWs from accessing HTC services [36, 46, 47]. On the other hand, when the services are well organised, they respond to the needs of the FSWs thus potentially improving access to HTC through improving both privacy and waiting time [48]. For instance, studies conducted in the Democratic Republic of Congo and Mozambique utilising night clinics placed at locations where FSWs frequent, led to improved access to HTC and greater service satisfaction among FSWs [48, 49].

### Affordability of HIV testing and counselling services

Fee for services, transport and other opportunistic costs can deter FSWs from accessing HTC services. Three studies reported that HTC services were free, at least in public health facilities, outreaches and campaigns [36, 44, 48]. One study indicated that FSWs are approximately four times more likely to go for HTC if the services are offered at a low cost or free (RDS adjusted % 4.4 [95%CI 1.7–7.7]) [46]. Even though HTC services were offered for free in most settings, FSWs complained of opportunity costs such as transport, long waiting time and bribes/tips requested by the HCWs as barriers to the uptake of these services [39, 46–48].

### Appropriateness of HIV testing and counselling services

The outcomes of the encounter with HTC service delivery point influences possibilities for further services use. This depends on the nature of the HTC service provided in meeting the needs and expectations of the FSWs. Studies document diverse reactions by the FSWs upon encounter with the HTC service providers. For example, some studies reported stigma, discrimination and poor attitudes of the health care workers (HCWs) towards FSWs [39, 40, 45]. These negative attitudes ranged from judgmental treatment, blaming, scolding, name calling, delaying services and at times even denying HTC to FSWs. The disrespectful behaviour was mostly shown by the HCWs from public health facilities [46, 48]. Further, unfriendly guidelines and policies such as preconditions set to access public health facilities, e.g. demand for Identity Cards, and the requirement to bringing sexual partners bared FSWs from using HTC services [36, 46, 48].

To increase access and uptake of HTC, some respondents preferred separate dedicated services for FSWs [39, 48]. For instance, studies conducted in Zimbabwe and the Democratic Republic of Congo reported preference for HTC offered by dedicated night clinics [47–49]. Increased client satisfaction was associated with the availability of tests and reagents, improved privacy and timely services as well as good reception and non-discrimination by the HCWs [47, 48]. However, some papers indicated that stand-alone night clinics might also pose a risk of stigma and discrimination to those seen entering the site [48]. Therefore, addressing the quality of care provided in the public health facilities through training the HCWs, improving privacy and facility organisation may also be needed to facilitate access to HTC services among FSWs [39, 48].

## Discussion

The findings of this review show that factors related to approachability, acceptability, availability, affordability and appropriateness of the services are crucial in influencing access to HTC services among FSWs in sub-Saharan Africa. The present review shows that factors related to approachability include, awareness on availability HTC services, and attitudes about testing and HIV risk perception. Evidence from this review shows that lack of awareness about availability, low perceptions about the importance of HTC (for HIV prevention), and low HIV risk perception were the main factors hindering FSWs access to HTC services. The review also found that acceptability of the HTC services is influenced by FSW peers and venue/bar owners or managers. Availability, accommodation and appropriateness of the HTC services for FSWs include factors such as the location of the services, privacy, confidentiality and overall quality of the HTC services for the FSWs. Most studies reported that HTC services were offered for free. However, upfront payments required to access HTC services from private health facilities and other hidden costs (e.g. transport cost and time spent to travel to the facilities) were a hindrance to utilise HTC services provided by private facilities.

Approachability factors are fundamental in improving access to HTC among FSWs. Findings from the present review and studies conducted elsewhere [50, 51] show that knowledge on the importance of HTC, service locations and HIV risk perception can affect uptake of HTC among FSWs. However, there are conflicting findings on risk perception. While studies in our review showed that high-risk perception was associated with uptake of HTC, a study conducted in China showed that FSWs who perceived being at risk were less likely to test for HIV [50]. Possibly, whereas some FSWs with high HIV risk perception access HTC to know their status [35, 36, 43], others feared the negative consequences of HIV positive results [38, 39, 46, 52, 53]. Several other studies conducted outside SSA, have indicated that fear of receiving positive test results override the decision to undertake HIV testing [51, 52, 54, 55]. For instance, studies conducted in the United States of America, India and Thailand also report fear of positive test result as a barrier to the uptake of HTC among FSWs [51, 52, 54, 55]. In our review, FSWs were concerned not only about the stigma attached to being a sex worker but also stigma attached to having HIV after they are found to be HIV positive at HTC clinics [56, 57]. For FSWs having HIV, had serious financial implication since HIV positive FSWs were denied access to recreational facilities were they can solicit clients [35]. It is for these reasons that, some of the FSWs felt it was better-off to remain without knowing their HIV status [52]. Concerns about stigma and related discriminations may lead to low uptake of HTC among FSWs even when services are readily available.

Social support and existing local networks have an essential role in increasing HTC services acceptability among FSWs. Findings from the present review suggest that motivation and encouragement by FSW peers and other trained peers have an important role in building awareness and creating demand for HTC services among FSWs [35, 36, 39, 42, 43, 45, 49]. The trust built between FSW peers can be a potential influencing agent for the uptake of HTC. Our review has also shown that, social structures around sex work context can also coerce FSWs to undertake HTC. For example, a study conducted in Guinea Conakry indicated that recreational facility managers and owners urged FSWs to test and/or disclose their HIV status before they were permitted to work at their facilities [35]. Nonetheless, the WHO emphasises that HTC services should be free of coercion [16]. Therefore, pressuring the FSWs to undergo HIV testing is a violation of their privacy and autonomy rights. Such violations of human rights increase in settings where sex work is illegal [37, 57, 58]. In these settings, the perpetrators, including police, health care workers and FSWs’ clients, exploit the illegality of sex work to violate their rights [57]. These violations, therefore, usually go unreported since reporting them would mean reporting oneself for illegal conduct [37, 46, 57].

Findings from the current review also show that availability, location and services organisation affected access to HTC among FSWs. This review found conflicting findings regarding how the proximity of health facilities impacted FSWs’ access to HTC. For example, some papers reported about FSWs who preferred to use nearby HTC service centres because of convenience and absence or small transport costs [36, 45, 47]. On the contrary, other FSWs preferred distant health facilities to avoid being seen by community members, and /or breach of confidentiality by health workers [35, 36, 44–46, 48]. Since findings on the role of proximity were overtly inconclusive, we think there is a need for a thorough understanding of how the proximity of health facilities facilitates or hinder access to HTC in the context of prevailing HIV and AIDS stigma.

Our review has also shown that FSWs were concerned with the quality of services offered by public health facilities [46, 48]. FSWs complained about abusive behaviour experienced from health workers at public health facilities, e.g. scolding, breach of confidentiality and name calling. Because of the fear about possible mishandling, FSWs visiting health facilities avoided disclosing their identity to the health care providers. Lack of disclosure of the FSWs' identity leads to a missed opportunity for HIV prevention interventions that are designed to target specifically FSWs.

To address FSWs concerns about the quality of health care services, some countries have opted to offer HTC through dedicated clinics – mostly managed by non-governmental organisation (NGOs) [47, 49]. These clinics were either exclusively for FSWs or included other key populations. Staffs in the dedicated clinics receive training to provide services that are tailored to the needs of the FSWs and other key populations where applicable. Unlike public health facilities, often dedicated clinics were attended by staff trained to provide services that are tailored to the needs of FSWs, and services were offered day and night [46, 48]. However, one study cautioned that at times dedicated clinics are ascribed to as being for sex workers or for the people living with HIV [48]. Because of the fear of this ascription FSWs may shy away from visiting dedicated clinics which are isolated from other health services. Probably, to address this concern, the dedicated clinics should be hosted or integrated with the infrastructure of the health facilities offering health services to the general populations.

For the program implementers and policymakers, there may also be concerns regarding the sustainability of the dedicated clinics, since they are fully or partially funded and managed by resources from external sources. Moreover, these clinics are built to improve project yields, and are usually few and located in settings with high numbers of FSWs. For this reason, they may not be equitably distributed in the community. It is therefore imperative that the public health facilities also improve their services to ensure equitable access to HTC among FSWs. Studies show that sensitivity training to HCWs has the potential to minimise stigma and discrimination at the health care settings [18, 59]. The training provided to the HCWs should also be coupled with job aides, supportive supervision and mentorship [18]. Ending stigma and discrimination, however, needs multi-sectoral effort, from the family, community and the government. Therefore, policies that protect high-risk populations against stigma and discrimination, and promote equitable access to HIV care and prevention services should be formulated.

This review has identified important findings within the available literature that could improve our understanding of the factors affecting access to HTC among FSWs in SSA. The use of a client-centred theoretical framework proposed by Levesque and colleagues has enhanced a thorough review of the factors affecting access to HTC among FSWs in SSA. HTC being a crucial step towards early initiation of care and treatment services which reduces further transmission of HIV [60], these findings can help programmers and policymakers in improving service provision to realise equitable access to HTC. This review has some limitations: First, only published literature was included. Although authors conducted hand-search of relevant references from included articles, insight from grey literature has not been included. Second, we used three databases- MEDLINE, POPLINE and Web of Science (WoS) in our search. This may have missed some literature from other databases. However, a systematic review on ideal database combinations for article searches suggested that the inclusion of MEDLINE and WoS databases alone can lead to the overall recall of about 85% [61]. Third, included studies were only those published in the English language, thus may have missed articles published in other languages used in SSA. Fourth, as the majority of publications came from ethnographic qualitative studies, our interpretation of the results may lack powers for broader generalizability. However, literature suggests that consistent findings across studies with sub-optimal risk of bias and generalizability may suggest the wider applicability of the findings [62]. Hence, we used the conceptual framework to narratively synthesise findings spanning from cross-sectional qualitative to longitudinal quantitative studies to improve the understanding of determinants of HTC uptake among FSWs in SSA.

## Conclusion

Given the important role that FSWs play in HIV transmission, targeting them with HIV prevention programs can have a potential to lower disease burden and decrease HIV transmission in the general population. HTC is recognised as a critical gateway to optimise delivery of prevention, care and treatment services. This systematic review reveals significant barriers and facilitators to HTC among FSWs in SSA.

Evidence from the current systematic review reveals that increasing awareness of the importance of HTC, peer support, availability and organisation of HTC services including flexible working hours have the potential to improve uptake of HTC among FSWs. On the contrary, long waiting time, lack of privacy, breach of confidentiality, stigma and discrimination impede FSWs’ uptake of HTC services. Stigma and discrimination, both in the community and at health facilities, emerged as important hindrances to accessing HTC among FSWs.

Adopting measures to address these barriers is necessary to improve uptake of HTC and other HIV prevention services among FSWs. A call by WHO for the national governments to decriminalise sex work, eliminate unjust application of non-criminal laws, and enact anti-discriminatory laws [20] may help to attract marginalised groups such as FSWs to seek HIV prevention services including HTC. To counteract stigma and discrimination at health system level, sensitivity training and education to all health care workers have proven to be effective and may enable provision of services that respond to specific needs of FSWs [20]. Since from a human rights perspective health is a fundamental right to all human being, it is imperative for program planners to encourage community dialogues and advocacy to promote a social environment that encourages marginalised populations such as FSWs to access HIV care and prevention services beings [63].

The evidence showed that lack of awareness of the importance of HTC and location of HTC services, fear of breach of confidentiality due to lack of privacy and confidentiality at health facilities, impeded FSWs to access HTC services. Concerns about breach of confidentiality (i.e. disclosure of HIV status to other community members), are reinforced and perpetuated by pervasive stigma toward people living with HIV. This review also underscores the role discriminatory norms toward sex work play in deterring FSWs to seek HTC services from public health facilities. It is therefore essential for HIV control programs to address conditions that amplify HIV stigma and discriminatory social norms against FSWs if we are to achieve equitable access to HTC among FSWs.

## Additional files

Additional file 1: PRISMA 2009 Checklist. (DOC 62 kb)

Additional file 2: PubMed illustrative search strategy. This file shows an illustrative search strategy as conducted on MEDLINE database (DOC 33 kb)

Additional file 3: Quality appraisal tool. (DOCX 20 kb)

## Abbreviations

FSWs: Female sex workers
HCWs: Health care workers
HTC: HIV testing and counselling
PROSPERO: Prospective Register of Systematic Reviews
RDS: Respondent-driven sampling
SSA: Sub-Saharan Africa
UNAIDS: Joint United Nations Programme on HIV/AIDS
WHO: World Health Organization
