# Supplementary material for: Determinants of access to HIV testing and counselling services among female sex workers in sub-Saharan Africa: a systematic review
Source: BMC Public Health. 2019 Jan 5;19:15. doi: 10.1186/s12889-018-6362-0 (PMC6321716; doi:10.1186/s12889-018-6362-0)
Supplement: Supplementary file 2 — PubMed illustrative search strategy. This file shows an illustrative search strategy as conducted on MEDLINE database (DOC 33 kb) [file 12889_2018_6362_MOESM2_ESM.doc]

**PubMed illustrative Search strategy**

**TOPIC: HIV**

("hiv"[MeSH Terms] OR "hiv"[All Fields]) OR (("hiv"[MeSH Terms] OR "hiv"[All Fields]) AND NEXT[All Fields] AND ("infection"[MeSH Terms] OR "infection"[All Fields])) OR ("hiv-1"[MeSH Terms] OR "hiv-1"[All Fields] OR "hiv 1"[All Fields]) OR ("hiv-2"[MeSH Terms] OR "hiv-2"[All Fields] OR "hiv 2"[All Fields]) OR (("humans"[MeSH Terms] OR "humans"[All Fields] OR "human"[All Fields]) AND NEXT[All Fields] AND ("immunologic deficiency syndromes"[MeSH Terms] OR ("immunologic"[All Fields] AND "deficiency"[All Fields] AND "syndromes"[All Fields]) OR "immunologic deficiency syndromes"[All Fields] OR "immunodeficiency"[All Fields]) AND NEXT[All Fields] AND ("viruses"[MeSH Terms] OR "viruses"[All Fields] OR "virus"[All Fields])) OR (acquired[All Fields] AND NEXT[All Fields] AND ("immunologic deficiency syndromes"[MeSH Terms] OR ("immunologic"[All Fields] AND "deficiency"[All Fields] AND "syndromes"[All Fields]) OR "immunologic deficiency syndromes"[All Fields] OR "immunodeficiency"[All Fields]) AND NEXT[All Fields] AND ("syndrome"[MeSH Terms] OR "syndrome"[All Fields])) OR (("hiv"[MeSH Terms] OR "hiv"[All Fields]) AND NEXT[All Fields] AND type[All Fields] AND 1[All Fields]) OR (("hiv"[MeSH Terms] OR "hiv"[All Fields]) AND NEXT[All Fields] AND type[All Fields] AND 2[All Fields])

AND

**POPULATION: Female sex workers**

(("women"[MeSH Terms] OR "women"[All Fields]) AND NEXT[All Fields] AND who[All Fields] AND NEXT[All Fields] AND sell[All Fields] AND NEXT[All Fields] AND ("sex"[MeSH Terms] OR "sex"[All Fields])) OR ("sex workers"[MeSH Terms] OR ("sex"[All Fields] AND "workers"[All Fields]) OR "sex workers"[All Fields] OR "prostitute"[All Fields]) OR (("female"[MeSH Terms] OR "female"[All Fields]) AND NEXT[All Fields] AND ("sex"[MeSH Terms] OR "sex"[All Fields]) AND NEXT[All Fields] AND worker[All Fields]) OR FSW[All Fields]

AND

**INTERVENTION: HIV testing**

(testing[All Fields] AND NEXT[All Fields] AND ("counselling"[All Fields] OR "counseling"[MeSH Terms] OR "counseling"[All Fields])) OR (voluntary[All Fields] AND NEXT[All Fields] AND testing[All Fields]) OR ("diagnosis"[Subheading] OR "diagnosis"[All Fields] OR "screening"[All Fields] OR "mass screening"[MeSH Terms] OR ("mass"[All Fields] AND "screening"[All Fields]) OR "mass screening"[All Fields] OR "screening"[All Fields] OR "early detection of cancer"[MeSH Terms] OR ("early"[All Fields] AND "detection"[All Fields] AND "cancer"[All Fields]) OR "early detection of cancer"[All Fields]) OR ("diagnosis"[Subheading] OR "diagnosis"[All Fields] OR "diagnosis"[MeSH Terms]) OR testing[All Fields] OR detection[All Fields] NOT ("early detection of cancer"[MeSH Terms] OR ("early"[All Fields] AND "detection"[All Fields] AND "cancer"[All Fields]) OR "early detection of cancer"[All Fields] OR ("cancer"[All Fields] AND "screening"[All Fields]) OR "cancer screening"[All Fields])

AND

**CONTEXT**:

"africa south of the sahara"[MeSH Terms] OR ("africa"[All Fields] AND "south"[All Fields] AND "sahara"[All Fields]) OR "africa south of the sahara"[All Fields] OR ("sub"[All Fields] AND "saharan"[All Fields] AND "africa"[All Fields]) OR "sub saharan africa"[All Fields]
