# Supplementary material for: Determinants of access to HIV testing and counselling services among female sex workers in sub-Saharan Africa: a systematic review
Source: BMC Public Health. 2019 Jan 5;19:15. doi: 10.1186/s12889-018-6362-0 (PMC6321716; doi:10.1186/s12889-018-6362-0)
Supplement: Supplementary file 3 — Quality appraisal tool. (DOCX 20 kb) [file 12889_2018_6362_MOESM3_ESM.docx]

**Additional file 3: Quality assessment tool**^[[1]](#footnote-1)^

| **Item** | **Well described** | **Moderately described** | **Poorly described** | **Unclear** |
| --- | --- | --- | --- | --- |
| Q1. Are the eligibility criteria and study context clearly described? |  |  |  |  |
| Q2. Are the methods fully described to allow replication?  (This includes details on coding, saturation and refusal rates) |  |  |  |  |
| Q3. Are the recruitment strategies appropriate for the study design used? |  |  |  |  |
| Q4. Are the results well described, accurate and consistent with the data collected?  (This includes the use of quotes, confidence intervals and statistical significance) |  |  |  |  |
| Q5. Are the results valid? (Design, objectives, bias minimisation strategies) |  |  |  |  |

**Average Quality Score**

| **Rate** | **Score** |
| --- | --- |
| High quality | ≥ 70% |
| Medium quality | 40 – 69% |
| Low quality | < 40% |

1. Items on this tool have been adapted from the Cochrane Collaboration qualitative study critical appraisal tool, the consolidated criteria for reporting qualitative studies (COREQ) tool and the strengthening reporting of observational studies in epidemiology (STROBE) tool. [↑](#footnote-ref-1)
